# Predictors of Persistent Participation in Youth Sport: A Systematic Review and Meta-Analysis

**DOI:** 10.3389/fpsyg.2022.871936

**Published:** 2022-05-27

**Authors:** Meng Zhang, Xiao-Chun Wang, Bin Shao

**Affiliations:** ^1^Institute of Physical Education, Huzhou University, Zhejiang, China; ^2^School of Physical Education and Training, Shanghai University of Sport, Shanghai, China; ^3^Institute of Foreign Studies, Huzhou University, Zhejiang, China

**Keywords:** factors, meta-analysis, persistent participation, sport, systematic review

## Abstract

There are many factors affecting decisions to persistent participation in sports and various approaches have been used to frame these antecedents. The aim of this paper was to systematically review and quantify the primary factors of persistent participation and to assess their respective strengths of association with persistent participation in youth sport. Adhering to the Preferred Reporting Items for Systematic Reviews and Meta-Analyzes (PRISMA) guidelines, a comprehensive search was implemented on 31st December 2021 in five databases and meta-analytic procedures were applied to data from studies meeting inclusion criteria. The results revealed that sports enjoyment (meta *r* = 0.45, 95%CI [0.42, 0.49]) was highly correlated with persistent intention, while persistent intention (SMD = 1.13, 95%CI [0.70, 1.56]) was highly correlated with persistent behavior. In addition, parental support, coach support, peer support, basic psychological needs and sports competence were the primary factors associated with persistent intention and persistent behavior, respectively. This study identified the key factors related to persistent participation and provide a complete understanding of children and adolescents' decisions to continue their participation in organized sports. Referring to the key factors, it can provide information for sports clubs and policy makers to develop strategies to increase youth participation in organized sport.

**Systematic Review Registration:**

PROSPERO, identifier: CRD42021229397.

## Introduction

Sport is defined as a structured, goal-oriented, competitive and contest-based form of physical activity. A growing body of studies have confirmed that persistent sports participation not only has a positive effect on the skills, physical development and social adaptation of adolescents, it also plays a significant role in reducing the risk of chronic diseases, cancer and obesity and in preventing psychological problems (Lindwall et al., 2014; Morris et al., 2019). In contrast to individuals who do not participate in sports, persistent participants experience less psychological difficulties and higher quality of life related to health (Vella et al., 2014a,b). In addition, exercise in adolescence increases the likelihood of physical activity in adulthood, thereby enabling the maintenance of a lasting and beneficial effect on their physical and mental health (Huotari et al., 2011). Despite these potential benefits, young athletes may be struggling with the decision to quit sports. Studies indicate that ~20–50% of young athletes between the ages of 10 and 17 dropout in sports each year (Balish et al., 2014). In Sweden, 77% of children aged 6–12 participate inorganized sports. However, between the ages of 13 and 25, this proportion dropped to 41% (Eliasson and Johansson, 2021). The portion of this dropout may reflect that the teenagers in the sample are trying to transfer between different sports, or quit sports in order to enter other fields such as music, STEM classes, etc. However, some withdrawal from sports may reflect dissatisfaction or negative experience. Sport dropout among young people has become such a common phenomenon in the world that the research on the behavior of persistent sports participation has been given more attention to by many clubs and researchers (Monteiro et al., 2018a; Eime and Harvey, 2019; Silva et al., 2019). Identifying the influencing factors of persistent sports participation in youth becomes more important.

From a measurement standpoint, dropout was primarily assessed on whether youth participants registered for their sport in subsequent seasons (Balish et al., 2014; Crane and Temple, 2015). Using registration in the subsequent season provides a minimalist view of dropout. Therefore, persistent participation is defined as participants go on their sport in the following season. The duration shall be at least 1 year. Many studies have attempted to explain sport persistence and dropout in relation to participants' underlying psychological characteristics, and various theories have been applied to frame this phenomenon. In previous studies, self-determination theory (SDT), achievement goal theory (AGT) and theory of planned behavior (TPB) are highly appropriate conceptual framework from which to study sport persistence and dropout. For example, Gardner et al. (2016) explored the antecedents of enjoyment and intention to continue in youth sports based on the theory of the AGT. Their study indicated that the social climate profiles were linked with intention to continue through enjoyment and the positive coach relationship quality profiles were relatively higher levels of enjoyment and intention to continue (Gardner et al., 2016). Joesaar and Hein (2011) integrated the AGT and SDT theories to confirm that youth athletes' task-involving peer motivational climate and intrinsic motivation predict sport persistence among the athletes (Joesaar et al., 2011). Gucciardi and Jackson (2015) integrated the theories of planned behavior (TPB) and basic psychological needs (BPN) to identify factors associated with young adults' continuation in organized sport. The results indicated that the satisfaction of basic psychological needs, intention and perceived behavioral control predicted sport continuation (Gucciardi and Jackson, 2015). In addition, researchers explored other factors of persistent participation in youth sports, such as demographic, biological, psychological, cultural, environmental (Boiche and Sarrazin, 2009; Bouffard, 2017; Wendling et al., 2018; Soares et al., 2020). Many cross-sectional and longitudinal studies have also shown that persistent sports participation is associated with higher perceived competence, self-esteem, and better emotional and social adaptation (Duda, 2013; Smith et al., 2016).

Although numerous studies identify the factors of persistent participation in youth sports, few researchers have conducted systematic review and meta-analysis of available articles to my knowledge. Due to the different research perspectives and theories adopted by researchers, the understanding of persistent participation in youth sports also differs, making it difficult to fully reflect the influencing factors of persistent participation. The strength or significance of some factors are also inconsistent in different articles. Therefore, it is essential to analyze the factors associated with persistent participation in youth sports through a systematic review and meta-analysis. The aims of this study are as follows: (a) to systematically review the factors of persistent sports participation in the empirical papers and (b) to assess their respective strengths associated with persistent participation in organized sport through the method of meta-analysis. This study can provide a complete understanding of children and adolescents' decisions to continue or discontinue their participation in organized sports. An overview of these factors can provide information that is useful for sports clubs and policy makers for developing new interventions to increase participation in organized sport among these adolescents.

## Methods

The present review was register on the international platform of PROSPERO (Booth et al., 2012) (registration number: CRD42021229397) and was reported in line with the Preferred Reporting Items for Systematic Reviews and Meta-Analysis (PRISMA) (Moher et al., 2015).

### Search Strategy

A comprehensive search was implemented on 31st December 2021 in five databases: Web of Science, PubMed, PsycINFO, SPORT Discus and ScienceDirect. Secondary literature was screened in the reference lists of included articles. To reduce the risk of removing relevant literature in the initial e-journal search, the author used broad search terms and rationalized the excluded terms in advance to reduce the risk of removing relevant literature in the initial (Gledhill et al., 2017). The search items focused on three key elements: (1) study population; (2) the context; and (3) outcome measure. The Boolean search terms were seen in Table 1.

**Table 1:** Search terms used for systematic review.

**Database**	**Search terms**
Web of science 504	TI = (sustained or prolonged or maintained or continu* or persist*) AND TS = (sport* or athlet*) AND TS = (child* or adolescen* or youth or teenager)
PubMed 761	((sustained[Title] OR prolonged[Title] OR continu*[Title] OR maintained[Title] OR persist*[Title]) AND (sport* OR athlet*[MeSH Terms])) AND (child* OR adolescen* OR youth OR teenager[MeSH Terms])
PsycINFO 100	TI (sustained OR prolonged OR continu* OR maintained OR persist*) AND AB (sport* OR athlet*) AND AB (child* OR adolescen* OR youth OR teenager)
SPORT Discus 125	TI (sustained OR prolonged OR continu* OR maintained OR persist*) AND AB (sport* OR athlet*) AND AB (child* OR adolescen* OR youth OR teenager)
ScienceDirect 215	TI (sustained OR prolonged OR continu* OR maintained OR persist*) AND AB (sport* OR athlet*) AND AB (child* OR adolescen* OR youth OR teenager)

**Figure 1:**
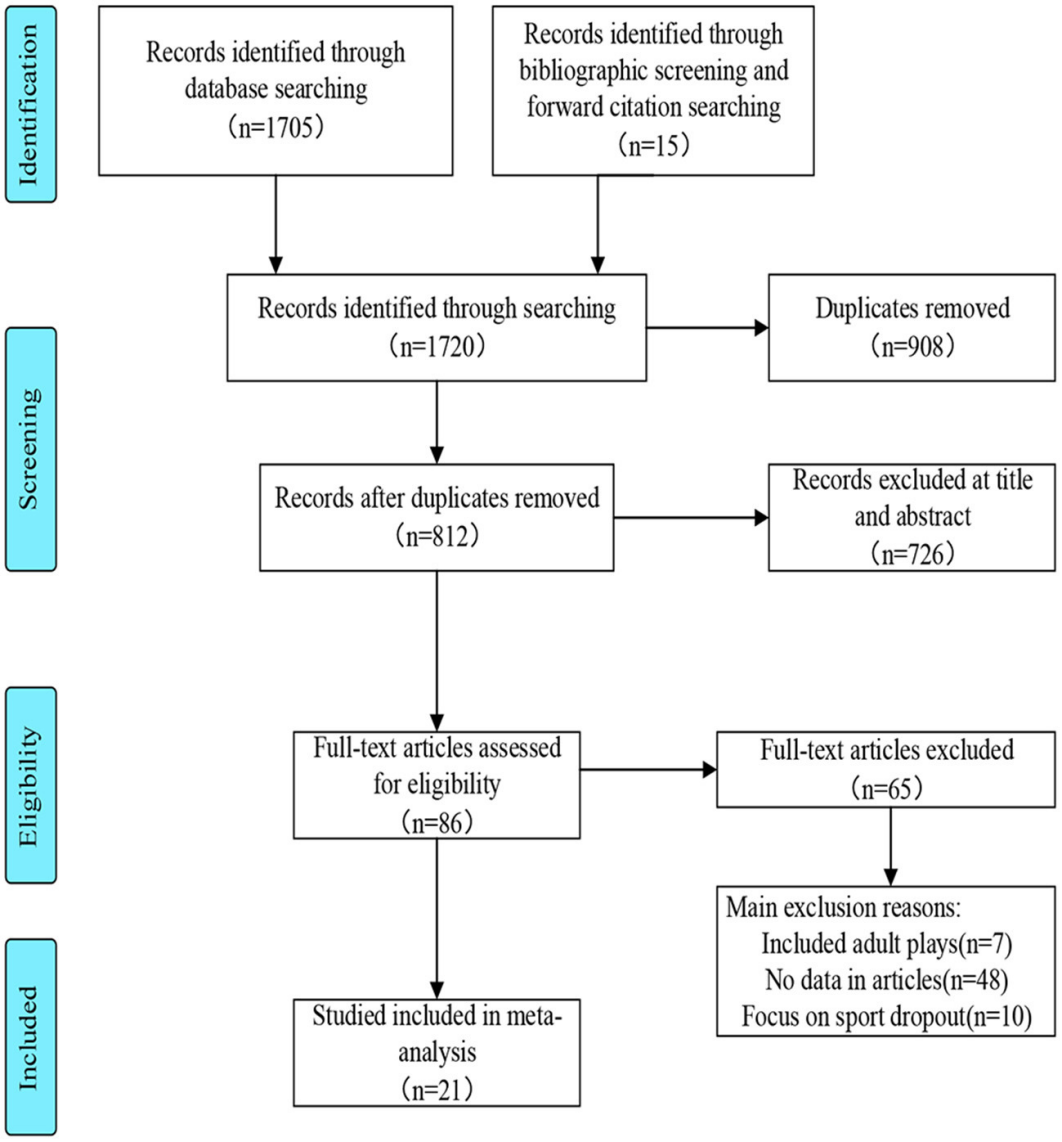
Flow diagram.

The flow diagram (Figure 1) summarizes the process for identification and selection of eligible studies. A total of 1,705 studies were searched from the electronic database, including WOS, PubMed, PsyCINFO, SPORT Discus, and Science Direct. Another 15 articles were identified through back referencing. All data were exported to EndNote X9 software. A total 908 duplicates were automatically removed through the Endnote X9 software. Following the title and abstract screening of the remaining records, 726 studies were removed for irrelevance. The full text of the remaining 86 articles was examined by two independent reviewers, resulting in 65 articles being excluded against the inclusion criteria. Finally, 21 articles were included for systematic review.

### Inclusion and Exclusion Criteria

Inclusion criteria: (1) Empirical studies pertaining to intention or behavior of persistent participation in sports; (2) Observational studies including cross-sectional or longitudinal research; (3) Contained correlation coefficient *r* or other effect values that could be converted into correlation coefficients, such as beta coefficient β; (4) The participations were children and/or adolescents aged between 5 and 19 years; (5) The extracted factors were mentioned three or more in papers. Studies were excluded if they: (1) were written in non-English; (2) did not contain original data or statistical analysis and (3) were reviews, dissertations or conference papers.

### Data Extraction

Two independently reviewers examined the full-text of the remaining 21 studies against the inclusion criteria and independently extracted and cross-checked them. All data were saved in an MS Excel spreadsheet. A data extraction form was used to obtain the authors, year of publication, country, research types, average age, sample size and related factors. Of the 21 articles included, 9 were cross-sectional studies and twelve were longitudinal studies. In the cross-sectional studies, the outcome variable was the intention of persistent participation, and the results provided the correlation coefficient *r* or β. In the longitudinal studies, the outcome variable was the behavior of persistent participation, and the results provided the mean and standard deviation of the different factors between persistent participation and sports dropout.

The Comprehensive Meta-Analysis (CMA) Version 3.0 software developed was used for meta-analysis and calculated the effect sizes and 95%CI. In the longitudinal studies of data analysis using ANOVA or *t*-test, the standard mean difference (SMD) and 95% confidence intervals (CIs) were calculated to evaluate the difference of all factors between persistent and dropout behaviors (Gillett, 2003). In the cross-sectional studies, the meta *r* was selected as the effect size to evaluate the relationship between different factors and persistent participation intention (Rosenthal and Dimatteo, 2001). When a regression β coefficient was reported, it would be converted into correlation *r* according to the formula recommended by Brown (*r* = β + 0.5λ, where λ equals 1 when β is non-negative and 0 when is negative) (Peterson and Brown, 2005). Based on the *r* and the sample size, the meta r was calculated using by CMA 3.0 software.

### Assessment of Study Quality

A quality appraisal was performed to minimize bias and improve the reliability of our findings. Two reviewers (MZ and XCW) independently assessed the quality of each study through the Quality in Prognosis Studies (QUIPS) checklist, which includes 6 domains: study participation, study attrition, prognostic factor measurement, outcome measurement, study confounding, statistical analysis and reporting (Hayden et al., 2006, 2013) (Supplementary Table 2). According to the evaluation criteria of the QUIPS, if any of the six domains was high risk, the quality was rated as “high” risk. Meanwhile, if none of the six domains was high risk or at least four domains were low risk, the quality was rated as “low” risk; Any cases other than these two rules were rated as “moderate” risk (Rabiee et al., 2021). Disagreements were discussed in a consensus meeting and agreement was reached by consulting a third reviewer (BS).

Heterogeneity among the studies was examined through a *Q*-test followed by the *I*^2^ statistic (Higgins et al., 2003). If *I*^2^ ≤ 50% and *P* > 0.05 in the *Q*-test, the heterogeneity was considered as not important and the fixed-effects model was performed. On the contrary, if *I*^2^ > 50% and *P* < 0.05 in the *Q*-test showed substantial heterogeneity, then the random-effects model was performed. The sensitivity was examined by comparing the difference in the effect sizes between the fixed-effects model and the-random effects model. The results are reliable if no difference was observed.

Assessment of publication bias was performed with Nfs (Fail safe number). The higher the Nfs value, the more unpublished studies needed to reverse the meta-analysis results, which meant that the publication bias was smaller and the meta-analysis results were more stable. According to the recommendation, no publication bias would occur if the value of Nfs was greater than 5*k* + 10 (*k* ≥ 3; *k* refers to the number of included studies).

## Results

### Overview of the Studies

Tables 2, 3 show that 21 articles (published from 2001 to 2021) were included, with a total sample size of 1,199. The surveyed countries included the USA (19.04%), Australia (19.04%), Spain (14.29%), Portugal (4.76%), Iran (4.76%), Finland (9.52%), France (14.29%), Estonia (9.52%), and Canada (4.76%). Among the 21 articles retained, 9 were cross-sectional studies (42.86%) and 12 were longitudinal studies (57.14%). The motivation theories adopted in these articles were mainly AGT and SDT. In the cross-sectional studies, the major outcome was persistent intention, which was measured in the form of questionnaire, such as PMCSQ, SMS, SCM and so on (seen in the Table 2). There were eight factors mentioned in included articles, including sports enjoyment, parental support, coach support, peer support, basic psychological needs, sports competence, task-involved climate and task orientation. In addition, 12 longitudinal studies were on persistent participation behavior, which compared the differences between several factors between persistent participation and dropout in sports. In these studies, the outcome was encoded with 1 (indicated persistent participants) and 0 (indicated dropouts). The duration of the longitudinal study ranged from 8 to 24 months, with an average of 14 months. There were 18 factors were mentioned in included articles, including: task-involved climate, ego-involved climate, basic psychological needs, persistent intention, intrinsic motivation, introjected regulation, amotivation, identified regulation, external regulation, parental support, coach support, peer support, sports competence, task orientation, ego orientation, years of involvement, amount of training and sports enjoyment.

**Table 2:** Basic characteristics of cross-sectional studies.

**No**.	**Study**	**Country**	**Age**	**Sample**	**Sports**	**Theory**	**Tools**	**Factors related to persistent intention**
1	Alvarez (2012)	Spain	14.77	370	soccer	SDT; AGT	PMCSQ-2; SMS	Task-involved climate (β = 0.10), Competence needs (β = 0.11), Autonomy needs (β = 0.16), Relatedness needs (β = 0.06)
2	Atkins (2013)	USA	12.7	227	volleyball, track, basketball, soccer	N/A	PDPR; MCYSQ; PSDQ; SCM	Sports enjoyment (β = 0.40), Sport competence (β = 0.1), Sport enjoyment (β = 0.34)
3	Atkins (2015)	USA	13.8	405	football, basketball, golf, hockey, swimming	AGT	MCYSQ; TEOSQ; PIMCQ-2; SCM	Peer support (β = 0.16), Parental support (β = 0.24), Task orientation (β = 0.51), coach support (β = 0.13), Sport competence (β = 0.20), Sport enjoyment (β = 0.48)
4	Gucciardi and Jackson (2015)	Australia	17.03	292	archery, golf, rugby triathlon, tennis, football, basketball	TPB; BPN	Not reported	Attitude, Perceptual behavior control
5	Gardner (2016)	Australia	13.03	313	soccer, netball, dancing, swimming	AGT	SCM; PPSS; SFQS; CARTQ	Parental support (*r* = 0.18), Coach support (*r* = 0.33), Friendship quality (*r* = 0.17), Peer acceptance (*r* = 0.16), sports enjoyment (*r* = 0.50), Social climate (β = −0.18), Friendship quality (β = −0.14)
6	Gardner (2017a)	Australia	13.03	327	soccer, netball, dancing	AGT	CNAAQ-2	Incremental beliefs (β = 0.02), Entity beliefs (β = 0.001)
7	Keshtidar (2017)	Iran	12.93	269	N/A	SDT; AGT	BRSQ	Task orientation (*r* = 0.21), Ego orientation (*r* = 0.24), Autonomous motivation (*r* = 0.37), Controlled motivation (*r* = 0.12)
8	Teixeira (2020)	Portugal	16.65	799	swimming	SDT	MCSYS; BPNES; BRSQ; PACES	Task-involved climate (*r* = 0.085), BPN satisfactory (*r* = 0.358), Sports motivation (*r* = 0.165), Sports enjoyment (*r* = 0.452)
9	Wekesser (2021)	USA	13.83	148	basketball, baseball	SDT	CARTQ	Coach support (β = 0.341)

**Table 3:** Basic characteristics of longitudinal studies.

**No**.	**Study**	**country**	**Age**	**Sample**	**Sports**	**Theory**	**Tools**	**Factors related to persistent participation**
1	Bars et al. (2009)	France	16.9	104	Judoka	AGT	SOGIRSQ; POSQ; GSE; PCJ	Coach support, parental support, peer support, task orientation, ego orientation, sport competence, self-esteem, physical condition, competition level
2	Calvo (2010)	Spain	14.3	492	Soccer	SDT	SMS	Amotivation, intrinsic motivation, identified regulation, introjected regulation, external regulation
3	Guillet et al. (2002)	France	16.06	253	Handball	SEM	SMS	Basic psychological needs, coach support, persistent intention, years of involvement, amount of training, sports commitment
4	Joesaar (2011a)	Estonia	12.7	659	Basketball, volleyball, soccer, swimming, badminton	AGT; SDT	MCYSQ; SCQ; SMS; BPNES	Coach support, parental support, basic psychological needs, task-involved climate, self-determination motivation
5	Joesaar (2011b)	Estonia	13.19	424	Basketball, soccer, volleyball	AGT; SDT	MCYSQ; BPNES; SMS	Task-involved climate, ego-involved climate, autonomy needs, competence needs, relatedness needs, intrinsic motivation
6	Pelletier (2001)	Canada	15.6	369	Swimming	SDT	SMS	Intrinsic motivation, identified regulation, introjected regulation, external regulation, amotivation, coach support
7	Rottensteiner (2015a)	Finland	15.09	1962	Soccer, ice hockey, basketball	AGT; SDT	PPCS; SMS	Task orientation, ego orientation, sport competence, persistent intention, self-determination motivation
8	Rottensteiner (2015b)	Finland	15.5	2235	Football, ice hockey, basketball	AGT	CART-Q; PMCSQ	Coach support, task-involved climate, ego-involved climate, years of involvement, amount of training, competition level
9	Ullrichfrench (2009)	USA	11.7	186	Soccer	SDT	SFQS; SEC; SMS	Parental support, peer support, sport competence, sports enjoyment, sports stress
10	Gardner (2017b)	Australia	13.01	373	N/A	FIT; TPB	SCM	Enjoyment, intention, perceived competence, parental support, coach support, peer support, friendship quality
11	Guzman (2012)	Spnish	15.3	857	Multiple sports	SDT	BPNS; SMS	Basic psychological needs, intention, perceived conflict
12	Sarrazin (2002)	Franch	14.07	335	Handball	SDT	SMS; PMCSQ	Intention, self-determination motivation

*PMCSQ-2, perceived motivational climate in sport questionnaire-2; SMS, sport motivation scale; SCMAM, social-cognitive model of achievement motivation; CNAAQ-2, conceptions of the nature of athletic ability questionnaire-version 2; MCYSQ, motivational climate in youth sport questionnaire; PSDQ, physical self-description questionnaire; SCM, sport commitment model; TEOSQ, task and ego orientation in sport questionnaire; PIMCQ-2, parental initiated motivational climate questionnaire 2; PPSS, perceived parental support scale; SFQS, sport friendship quality scale; CART-Q, coach athlete relationship questionnaire; BRSQ, behavioral regulation in sports questionnaire; MCSYS, motivational climate sport youth scale; BPNES, basic psychological needs exercise scale; BRSQ, behavioral regulation in sport scale; PACES, physical activity enjoyment scale; SOGIRSQ, significant others' goal-involving roles in sport questionnaire; POSQ, perception of success questionnaire; GSE, global self-esteem scale; PCJ, perceived competence in judo; BPNES, basic psychological needs in exercise scale; SCQ, sport climate questionnaire; PPCS, perceived physical competence scale; SFQS, sport friendship quality scale; SEC, sport enjoyment scale*.

### Heterogeneity

In the longitudinal studies, given the substantial heterogeneity of persistent intention (*I*^2^ = 89%), parental support (*I*^2^ = 61%), coach support (*I*^2^ = 95%), peer support (*I*^2^ = 82%), and intrinsic motivation (*I*^2^ = 98%), a random-effect model was used to analyze the difference between persistent participation and sports dropout. A fixed-effect model was used to analysis for basic psychological needs (*I*^2^ = 48%) and sports competence (*I*^2^ = 0%) as low heterogeneity was observed. In the cross-sectional studies, for coach support (*I*^2^ = 98%) and basic psychological needs (*I*^2^ = 92%), a random-effect model has used to analyze the correlation between factors and participation intention. A fixed-effect model was used to analysis for sports enjoyment, parental support, peer support and sports competence as low heterogeneity was observed.

### Sensitivity and Publication Bias

In the statistical process with a fixed-effects or random-effects model, no significant difference among effect sizes was observed after removing each article in turn, nor was 95%CI. The significance of all factors did not change. Hence, the sensitivity of the included article was low, and the result of the meta-analysis was more reliable. Tables 5, 6 showed that the Nfs of parental support, peer support and sport competence were low, indicating that more articles were needed to revise the results of meta-analysis. In addition, the Nfs of other factors were greater than the recommended values (5*k* + 10), indicating the absence of publication bias in these studies. The final assessment showed that 12 articles were low risk, five articles were moderate risk, and two articles were high risk (Table 4). A high risk of bias was awarded to two studies mainly because inclusion and exclusion criteria were failure to report, participants lost to follow-up were not adequately described, and important potential confounders were not accounted for in the analysis and selective reporting of results.

**Table 4:** QUIPS risk of bias assessment.

**Study**	**Study**	**Study**	**Factor**	**Outcome**	**Confounding**	**Statistical**	**Overall**
	**participation**	**attrition**	**measurement**	**measurement**	**measurement**	**analysis**	**quality**
Alvarez (2012)	Moderate	N/A^a^	Low	Low	N/A	Low	Low
Atkins (2013)	Low	N/A	Low	Low	Moderate	Low	Low
Atkins (2015)	Low	N/A	Low	Low	N/A	Low	Low
Gucciardi and Jackson (2015)	Moderate	N/A	Low	Low	Moderate	Low	Moderate
Gardner et al. (2016)	Low	N/A	Low	Low	Moderate	Low	Low
Gardner (2017a)	Low	N/A	Low	Low	Moderate	Low	Low
Keshtidar (2017)	Moderate	N/A	Moderate	Low	N/A	Moderate	Moderate
Teixeira (2020)	Moderate	N/A	Low	Low	N/A	Low	Low
Wekesser (2021)	Low	N/A	Low	Low	N/A	Low	Low
Bars et al. (2009)	Moderate	N/A	Low	Low	N/A	Moderate	Moderate
Calvo (2010)	Moderate	N/A	Low	Moderate	N/A	Moderate	Moderate
Guillet et al. (2002)	Moderate	N/A	High	Low	N/A	Low	High
Joesaar (2011a)	Moderate	N/A	Low	Low	N/A	Moderate	Moderate
Joesaar (2011b)	Moderate	N/A	Low	Low	N/A	Low	Low
Pelletier et al. (2001)	Moderate	N/A	Low	Low	Moderate	High	High
Rottensteiner (2015a)	Moderate	N/A	Low	Low	N/A	Low	Low
Rottensteiner (2015b)	Low	N/A	Low	Low	N/A	Low	Low
Ullrich-French (2009)	Low	N/A	Low	Low	Moderate	Low	Low
Gardner (2017b)	Low	N/A	Low	Low	Moderate	Low	Low
Guzman (2012)	Low	N/A	Low	Low	Moderate	Low	Low
Sarrazin et al. (2002)	Low	N/A	Low	Low	Moderate	Low	Low

a *N/A, Not applicable*.

**Table 5:** Meta-Analysis of factors related to persistent participation intention.

**Factors**	***k***	***I* ^2^**	***p***	**Model**	**Meta *r***	**95%CI**	***z***	***p***	**Nfs**	**Strength**
Sports enjoyment	4	47%	0.128	Fixed-effects Model	0.45	0.42–0.49	20.32	0.000	395	High
Basic psychological needs	3	92%	0.000	Random-effects Model	0.41	0.27–0.50	5.37	0.000	305	Medium
Coach support	4	98%	0.000	Random-effects Model	0.43	0.08–0.69	2.34	0.020	185	Medium
Parental support	3	0%	0.410	Fixed-effects Model	0.20	0.13–0.26	5.98	0.000	23	Low
Sport competence	3	0%	0.372	Fixed-effects Model	0.18	0.11–0.24	5.10	0.000	17	Low
Peer support	3	0%	0.933	Fixed-effects Model	0.17	0.10–0.23	5.02	0.000	17	Low

**Table 6:** Meta-Analysis of factors related to persistent participation behavior.

**Factor**	***k***	**I^2^**	***p***	**Model**	**SMD**	**95%CI**	***z***	***p***	**Nfs**	**Strength**
Coach support	5	95%	0.000	Random-effects Model	0.68	0.27–1.10	3.22	0.001	288	Medium
Peer support	3	82%	0.004	Random-effects Model	0.50	0.07–0.92	2.28	0.022	20	Medium
Parental support	4	61%	0.050	Random-effects Model	0.37	0.11–0.62	2.79	0.005	20	Low
Sport competence	3	0%	0.396	Fixed-effects Model	0.33	0.24–0.43	6.62	0.000	25	Low
Basic psychological needs	4	48%	0.123	Fixed-effects Model	0.29	0.21–0.38	6.81	0.000	43	Low
Intrinsic motivation	5	98%	0.000	Random-effects Model	0.74	0.18–1.31	2.58	0.010	322	Medium
Persistent intention	3	89%	0.000	Random-effects Model	1.13	0.70–1.56	5.12	0.000	246	High

### Meta-Analysis

According to the calculation results of meta *r*, six factors were significantly related to the intention to persist (shown in Table 5). Following the recommendation in Lipsey et al., the meta *r* < 0.25 represented weak correlation, 0.25 < *r* < 0.4 represented moderate correlation, and *r* > 0.4 represented high correlation (Lipsey and Wilson, 2000). The results showed that among the factors related to participation intention, sports enjoyment reached high correlation; basic psychological needs and coach support reached moderate correlation, parental support, peer support and sports competence reached low correlation.

By calculating the effect sizes (shown in Table 6), the results indicate that the seven factors had significant differences in terms of persistent participation and dropout, including: persistent intention, parental support, coach support, peer support, basic psychological needs, intrinsic motivation and sports competence. According to the recommendation of Cohen, the benchmarks of SMD = 0.3, 0.5, and 0.8 represent low, moderate and high effects, respectively (Field and Gillett, 2010; Thompson et al., 2018). The above results indicated that persistent intention reached a large effect; coach support, peer support and intrinsic motivation reached medium effects; parental support, basic psychological needs and sports competence reached small effects.

## Discussion

Although numerous studies identify the factors of persistent participation in youth sports, this review synthesizes the factors of persistent participation by using the methods of meta-analysis. Due to the different research perspectives and theories adopted by researchers, the understanding of persistent participation in youth sports also differs, making it difficult to fully reflect the influencing factors of persistent participation. The strength or significance of some factors are also inconsistent in different articles. In this review we summarized the primary factors of persistent participation and conducted a meta-analysis of available effect sizes to assess their respective strengths of association with persistent participation in youth sport. Our findings showed that sports enjoyment was highly correlated with persistent intention, while persistent intention was highly correlated with persistent behavior. In addition, parental support, coach support, peer support, basic psychological needs and sports competence were the primary factors associated with persistent intention and persistent behavior, respectively.

Consistent with previous studies, sports enjoyment and persistent intention were the two important variables for studying sports behavior (Quested et al., 2013; Gardner et al., 2016). Many researchers have also confirmed that sports enjoyment and intention were the key motivational processes that affected teenagers' persistent participation in sports, and lack of enjoyment was the main reason for dropout (Gardner et al., 2017). In the present study, sports enjoyment was highly correlated with persistent intention. However, among the factors of persistent behavior, the factor of sports enjoyment was not identified. The main reason for this finding was that among the 12 longitudinal studies, only two articles demonstrated the influence of sports enjoyment on persistent behavior. Hence, it did not meet the criteria of selection, which should be mentioned in more than two articles. There was a medium effect between intrinsic motivation and persistent behavior, but the extrinsic motivation including three forms of regulation had no significant effect. Although many researchers confirmed that intrinsic motivation and amotivation were the main factors that affect athletes' persistent participation in sports, the conclusions of the studies on the influence of different forms of extrinsic motivation on participation behavior were inconsistent. For example, Vallerand and Rousseau (2001) found that identity regulation was also an important part of athletes' performance, in addition to intrinsic motivation. Pelletier et al. (2001) also believed that a significant difference in identity regulation between persistent and dropout athletes existed, but no significant difference in introjected regulation.

Many researchers indicated that parental support, coach support and peer support were significantly correlated with persistent intention and behavior (Harwood et al., 2015; Monteiro et al., 2018b). In the current study, parental support weakly correlated with persistent intention and persistent behavior. The reason might be children with parental support tended to experience more enjoyment and intrinsic motivation, and thus, they were more likely to continue to participate (Fredricks and Eccles, 2002). However, as children grow older, parents' support gradually decreased because the learning tasks increased.

The relationship between coach support and persistent intention was moderately correlated along with the behavior of persistent participation. The factor of coach support was a key component of the motivational climate, which was characterized by a high sense of intimacy, commitment, complementarity and common orientation (Jowett and Ntoumanis, 2004). Many psychological outcomes in sports, including motivation and intention to participate, were related to coach support (Riley and Smith, 2011; Rocchi et al., 2017). However, low-quality coach-athlete relationships, including coach conflict, coach's control styles, lack of encouragement, and an overemphasis on victory were usually associated with sports dropout behavior (Gearity and Murray, 2011).

A significantly positive relationship between peer support and intention and behavior of persistent participation was also observed. In sports, young people regarded the perceived peer acceptance and friendship as one of the driving forces for persistent participation (Keegan et al., 2010). Ullrichfrench and Smith (2006) found that peer support was significantly correlated with persistent participation intention, and high-quality friendship could buffer negative results related to acceptance.

The relationship between basic psychological needs and persistent intention was moderately correlated but weakly correlated with persistent behavior. For example, Guillet et al. (2002) confirmed that persistent players perceived themselves as significantly more competent, more autonomous and more related to their team than dropout players. Joesaar et al. (2011) also supported the results in which dropout athletes' satisfaction with autonomy, competence and relatedness needs were lower than that of persistent athletes. According to the self-determination theory, only when the 3 basic psychological needs (competence, autonomy and relatedness) were satisfactory could athletes maintain the motivation of self-determination. On the contrary, uncertain motivation and incentive forms were promoted, which might lead to sports dropout (Sarrazin et al., 2002).

The results of the research on the relationship between sports competence and persistent participation were inconsistent. Athletes with better sports competence were generally believed to be more likely to continue to participate in sports, and the perceived competence was considered to be an important predictor of persistent behavior (Soderstrom et al., 2018). The comparative study of persistent behavior and sports dropout indicated that the score of sports competence of dropout athletes was significantly lower than that of persistent athletes. On the contrary, Bars et al. (2009) and Calvo et al. (2010) believed that sports competence was not an important predictor of persistent participation, and relatedness and autonomy might be more important than sports competence in explaining persistent participation or dropout behavior. The reason for the above inconsistency might be that different methods and tools were used to evaluate sports competence. For example, the tool with only 1 item used in Calvo's study might not be the best way to assess the sports competence of athletes.

In this study, among the included articles, although 2 ones studied years of involvement, meta-analysis was not carried out because they did not meet the criteria. In previous studies, years of involvement were significantly associated with persistent participation. Rottensteiner et al., 2015 suggested that persistent athletes appeared to show a higher level of competition, long years of participation and higher exercise frequency in sports than dropout athletes. However, Guillet et al. (2002) showed that although the years of involvement of persistent athletes were higher than those of dropout athletes, no difference in weekly exercise frequency could be observed between the two groups, and no significant correlation between years of involvement and persistent participation behavior. To sum up, not much evidence was found to prove the causal relationship between the years of involvement and persistent participation. In the future, the relationship between years of involvement and persistent participation behavior should be discussed further by expanding the sample of athletes.

## Conclusion And Limitations

The paper systematically reviews the different viewpoints and conclusions on the influencing factors of persistent participation in youth sports. By utilizing techniques of meta-analysis, this study synthesizes 6 factors related to the intention of persistent participation and seven factors related to the behavior of persistent participation in sports. It can provide a reference for researchers to further understand the relationship between influencing factors and persistent participation in youth sports. In addition, this study strictly follows the principle of meta-analysis to collect and analyse data, making the analysis and results more standardized and reliable. It not only enriches the research content of persistent participation in youth sports but also provides insights into future research in this field.

Although the collected articles come from many important databases, it is still incomplete because the conference database was not retrieved. Second, there are inherent limitations in meta-analysis methods and the software used. For example, the selected factors must be mentioned more than twice in papers, resulting in incomplete factors. In addition, the effect size of some factors is small because few articles and samples related to these factors were found, and the results need to be confirmed further by more articles. Finally, this study only includes the independent variables directly related to the dependent variables as influencing factors, and future research should further consider the effects of mediating or moderating variables and the interaction of independent variables.

## Data Availability Statement

The datasets presented in this study can be found in online repositories. The names of the repository/repositories and accession number(s) can be found in the article/Supplementary Material.

## Author Contributions

MZ draft almost the manuscript. X-CW did data collection and coding and data analysis. BS contributed to study design, supervision, and paper review. All authors contributed to the article and approved the submitted version.

## Conflict of Interest

The authors declare that the research was conducted in the absence of any commercial or financial relationships that could be construed as a potential conflict of interest.

## Publisher's Note

All claims expressed in this article are solely those of the authors and do not necessarily represent those of their affiliated organizations, or those of the publisher, the editors and the reviewers. Any product that may be evaluated in this article, or claim that may be made by its manufacturer, is not guaranteed or endorsed by the publisher.
