# Supplementary material for: Predictors of Persistent Participation in Youth Sport: A Systematic Review and Meta-Analysis
Source: Front Psychol. 2022 May 27;13:871936. doi: 10.3389/fpsyg.2022.871936 (PMC9196305; doi:10.3389/fpsyg.2022.871936)
Supplement: Supplementary file 2 [file Table_2.DOCX]

**Supplementary Table2 - QUIPS Guide for Assessing Risk of Bias**

| **Domains** | **Prompting Items for Consideration** | **Ratings** |
| --- | --- | --- |
| Study Participation | 1. Adequate participation in the study by eligible persons.  2. Description of the source population or population of interest.  3. Description of the baseline study sample.  4. Adequate description of the sampling frame and recruitment.  5. Adequate description of the period and place of recruitment.  6. Adequate description of inclusion and exclusion criteria. | **High bias:** The relationship between the PF and outcome is very likely to be different for participants and eligible nonparticipants  **Moderate bias**: The relationship between the PF and outcome may be different for participants and eligible non-participants  **Low bias**: The relationship between the PF and outcome is unlikely to be different for participants and eligible nonparticipants |
| Study Attrition | 1. Adequate response rate for study participants.  2. Description of attempts to collect information on participants who dropped out.  3. Reasons for loss to follow-up are provided.  4. Adequate description of participants lost to follow-up.  5. There are no important differences between participants who completed the study and those who did not. | **High bias**: The relationship between the PF and outcome is very likely to be different for completing and non-completing participants.  **Moderate bias**: The relationship between the PF and outcome may be different for completing and non-completing participants.  **Low bias**: The relationship between the PF and outcome is unlikely to be different for completing and non-completing participants. |
| Prognostic Factor  Measurement | 1. A clear definition or description of the PF is provided.  2. Method of PF measurement is adequately valid and reliable.  3. Continuous variables are reported or appropriate cut points are used.  4. The method and setting of measurement of PF is the same for all study participants.  5. Adequate proportion of the study sample has complete data for the PF.  6. Appropriate methods of imputation are used for missing PF data. | **High bias**: The measurement of the PF is very likely to be different for different levels of the outcome of interest.  **Moderate bias**: The measurement of the PF may be different for different levels of the outcome of interest.  **Low bias**: The measurement of the PF is unlikely to be different for different levels of the outcome of interest. |
| Outcome  Measurement | 1. A clear definition of the outcome is provided.  2. Method of outcome measurement used is adequately valid and reliable.  3. Method and setting of outcome measurement is the same for all study participants. | **High bias**: The measurement of the outcome is very likely to be different related to the baseline level of the PF.  **Moderate bias**: The measurement of the outcome may be different related to the baseline level of the PF.  **Low bias**: The measurement of the outcome is unlikely to be different related to the baseline level of the PF. |
| Study Confounding | 1. All important confounders are measured.  2. Clear definitions of the important confounders measured are provided.  3. Measurement of all important confounders is adequately valid and reliable.  4. The method and setting of confounding measurement are the same for all study participants.  5. Appropriate methods are used if imputation is used for missing confounder data.  6. Important potential confounders are accounted for in the study design.  7. Important potential confounders are accounted for in the analysis. | **High bias:** The observed effect of the PF on the outcome is very likely to be distorted by another factor related to PF and outcome.  **Moderate bias:** The observed effect of the PF on outcome may be distorted by another factor related to PF and outcome.  **Low bias:** The observed effect of the PF on outcome is unlikely to be distorted by another factor related to PF and outcome. |
| Statistical Analysis and Reporting | 1. Sufficient presentation of data to assess the adequacy of the analytic strategy.  2. Strategy for model building is appropriate and is based on a conceptual framework or model.  3. The selected statistical model is adequate for the design of the study.  4. There is no selective reporting of results. | **High bias**: The reported results are very likely to be spurious or biased related to analysis or reporting.  **Moderate bias**: The reported results may be spurious or biased related to analysis or reporting.  **Low bias**: The reported results are unlikely to be spurious or biased related to analysis or reporting. |

PF: prognostic factor
