# Supplementary material for: Predictors of Persistent Participation in Youth Sport: A Systematic Review and Meta-Analysis
Source: Front Psychol. 2022 May 27;13:871936. doi: 10.3389/fpsyg.2022.871936 (PMC9196305; doi:10.3389/fpsyg.2022.871936)
Supplement: Supplementary file 3 [file Table_3.docx]

**Supplementary Table 3 Data of the longitudinal study**

| Factor | Study | Persistent | | | Dropout | | |
| --- | --- | --- | --- | --- | --- | --- | --- |
|  |  | M1 | SD1 | n1 | M2 | SD2 | n2 |
| Partental support | Joesaa, 2011a | 6.02 | 1.00 | 320 | 5.8 | 1.05 | 339 |
|  | Bars, 2009 | 3.92 | 0.72 | 52 | 3.42 | 0.69 | 52 |
|  | Ullrich-French, 2009 | 3.53 | 0.99 | 115 | 3.425 | 0.92 | 33 |
|  | Gardner, 2017 | 4.23 | 0.65 | 247 | 3.86 | 0.82 | 26 |
| Coach support | Guillet, 2002 | 4.39 | 1.51 | 230 | 3.45 | 1.65 | 23 |
|  | Joesaar, 2011a | 5.49 | 1.09 | 320 | 5.35 | 1.15 | 339 |
|  | Bars, 2009 | 4.64 | 0.56 | 52 | 4.31 | 0.59 | 52 |
|  | Rottensteiner, 2015a | 5.66 | 1.16 | 1695 | 4.74 | 1.53 | 548 |
|  | pelletier, 201 | 15.86 | 1.26 | 194 | 14.26 | 1.08 | 175 |
| Peer support | Joesaar, 2011a | 5.06 | 1.03 | 320 | 4.84 | 1.03 | 339 |
|  | Bars, 2009 | 4.46 | 0.57 | 52 | 3.95 | 0.51 | 52 |
|  | Ullrich-French, 2009 | 3.39 | 0.48 | 115 | 3.19 | 0.48 | 33 |
| Basic psychological  needs | Joesaar, 2011a | 3.95 | 0.74 | 320 | 3.81 | 0.78 | 339 |
|  | Joesaar, 2011b | 3.96 | 0.73 | 282 | 3.74 | 0.77 | 142 |
|  | Calvo, 2010 | 7.08 | 1.31 | 314 | 6.76 | 1.35 | 178 |
|  | Guzmán, 2012 | 5.58 | 0.91 | 628 | 5.18 | 0.91 | 229 |
| Intrinsic motivation | Rottensteiner, 2015a | 3.5 | 0.71 | 1517 | 3.16 | 0.77 | 445 |
|  | Joesaar, 2011a | 5.73 | 1.02 | 320 | 5.47 | 1.12 | 339 |
|  | Joesaar, 2011b | 5.57 | 1.07 | 282 | 5.25 | 1.09 | 142 |
|  | Calvo, 2010 | 3.95 | 0.81 | 314 | 3.72 | 0.86 | 178 |
|  | pelletier,201 | 17.01 | 1.14 | 194 | 14.62 | 0.72 | 175 |
| Persistent intention | Rottensteiner, 2015 | 5.23 | 1.24 | 1695 | 4.19 | 1.56 | 548 |
|  | Gardner, 2017 | 4.68 | 0.60 | 247 | 3.54 | 1.24 | 26 |
|  | Sarrazin, 2002 | 3.86 | 1.91 | 261 | 1.96 | 1.25 | 74 |
| Sport competence | Rottensteiner, 2015 | 3.87 | 0.57 | 1517 | 3.68 | 0.67 | 445 |
|  | Bars, 2009 | 4.02 | 1.08 | 52 | 3.74 | 0.97 | 52 |
|  | Ullrich-French, 2009 | 2.81 | 0.55 | 155 | 2.48 | 0.59 | 31 |
