# Supplementary material for: Predictors of Persistent Participation in Youth Sport: A Systematic Review and Meta-Analysis
Source: Front Psychol. 2022 May 27;13:871936. doi: 10.3389/fpsyg.2022.871936 (PMC9196305; doi:10.3389/fpsyg.2022.871936)
Supplement: Supplementary file 4 [file Table_4.docx]

**Supplementary Table 4 Data of the cross-sectional study**

| Factor | Author | r | n |
| --- | --- | --- | --- |
| Sport competence | Atkins, 2013 | 0.10 | 227 |
|  | Atkins, 2015 | 0.20 | 405 |
|  | Ullrich-French, 2009 | 0.22 | 186 |
| Sport enjoyment | Atkins, 2013 | 0.34 | 227 |
|  | Gardner, 2016 | 0.50 | 313 |
|  | Teixeira, 2020 | 0.45 | 799 |
|  | Atkins, 2015 | 0.48 | 405 |
| Parental support | Gardne, 2016 | 0.18 | 313 |
|  | Atkins, 2015 | 0.24 | 405 |
|  | Ullrich-French, 2009 | 0.13 | 186 |
| Coach support | Gardner, 2016 | 0.33 | 313 |
|  | Atkins, 2015 | 0.13 | 405 |
|  | Pelletier, 2001 | 0.18 | 369 |
|  | Wekesser, 2021 | 0.84 | 148 |
| Peer support | Gardner, 2016 | 0.16 | 313 |
|  | Ullrich-French, 2009 | 0.19 | 186 |
|  | Atkins et al., 2015 | 0.16 | 405 |
| Basic psychological needs | Alvarez, 2012 | 0.33 | 370 |
|  | Teixeira, 2020 | 0.36 | 799 |
|  | Guzmán, 2012 | 0.53 | 857 |
